# Dedifferentiated chondrosarcoma of the pelvis: clinical outcomes and current treatment

**DOI:** 10.1186/s13569-018-0110-1

**Published:** 2018-12-14

**Authors:** Johnathan R. Lex, Scott Evans, Jonathan D. Stevenson, Michael Parry, Lee M. Jeys, Robert J. Grimer

**Affiliations:** 1 Royal Orthopaedic Hospital, Birmingham, B31 2AP UK; 2 Aston University, Birmingham, UK

**Keywords:** Chondrosarcoma, Dedifferentiated, Pelvis, Amputation, Limb-salvage, Sarcoma

## Abstract

**Background:**

Dedifferentiated chondrosarcomas (CS) are a high-grade variant of CS that confers a 5-year survival of around 10–24%. Dedifferentiated CS arising from the pelvis confers an even worse prognosis.

**Questions:**

(1) What is the prognosis of patients with dedifferentiated CS of the pelvis? (2) Do wide margins or type of surgical intervention influence outcome? (3) Does the use of adjuvant therapy affect outcome?

**Methods:**

Patients were retrospectively reviewed from a prospectively collated musculoskeletal oncology database from 1995 to 2016. Thirty-one cases of dedifferentiated CS arising from the pelvis were included. Wide margins were defined as greater than 4 mm. The mean age was 55.6 years (range 33 to 76 years) and there were 19 males (61.3%) and 12 females (38.7%).

**Results:**

The disease presented at a locally or systemically advanced stage in 13 patients (41.9%). Eighteen patients (58.1%) underwent surgery with curative intent. Overall survival at 12 months was 15.4% for patients treated with palliative intent and 50% for those treated with surgery. In the surgical group, there were higher rates of disease-free survival in patients who underwent hindquarter amputation and those who received wide surgical margins (p = 0.047 and p = 0.019, respectively). Those who underwent hindquarter amputation were more likely to achieve wide margins (p = 0.05). Time to recurrent disease (local or systemic) was always less than 24 months. No hindquarter amputation for recurrent disease resulted in disease-free survival. No patient who received adjuvant therapy for palliative or recurrent disease had disease control.

**Conclusions:**

Pelvic dedifferentiated CS often presents at an advanced local or systemic stage and confers a poor prognosis. Achieving wide surgical margins (> 4 mm) provided the highest rate of long-term disease-free survival. Failing to achieve wide margins results in rapid disease recurrence, conferring deleterious consequences.

## Introduction

Chondrosarcoma (CS) is a rare malignant bone tumour composed of cartilage matrix-producing cells. It is the second most common primary bone sarcoma with an incidence of 1 in 200,000/year. It may arise in the medullary cavity of bone (central CS) or secondary to a malignant transformation of a benign cartilage tumour [1]. The most important factor for guiding management and prognosis is determining the histological grade of the tumour. There is a high degree of inter-observer variability when determining histological grade [2]. Dedifferentiated CS is defined as one area of a lower grade cartilage tumour that lies directly adjacent to an area of high-grade non-cartilaginous sarcoma [3]. Only 10% of all CS dedifferentiate, which is fortunate as these high-grade tumours are associated with a 5-year survival of around 10–24% [1, 4, 5].

Chondrosarcomas, in general, are resistant to chemotherapy and conventional radiotherapy. Occasionally, short-term local control can be achieved but has no proven benefit on distant spread or overall survival [6–8]. Consequently, surgery remains the mainstay of treatment for dedifferentiated CS. Dedifferentiated CS arising from the pelvis is known to be a negative prognostic factor, further lowering the survival rate [9, 10]. For this disease, there is limited evidence defining the presenting disease stage, accuracy of pre-operative diagnosis and treatment factors influencing outcome [9].

Current treatment for pelvic dedifferentiated CS consists of either palliative, limb-salvage through pelvic resection with or without reconstruction, or limb-sacrifice with hindquarter amputation [5, 6]. Tumour excision with wide margins should provide the best prognosis although this is often difficult due to the proximity of vital structures. This association and the probability of achieving wide margins with limb-salvage has yet to be described in pelvic dedifferentiated CS.

We report our experience with dedifferentiated CS of the pelvis including diagnosis, survival and surgical outcomes. The questions we attempted to answer in this study were; (1) what is the prognosis of patients with dedifferentiated CS of the pelvis? (2) Do wide margins or type of surgical intervention influence outcome in patients for attempted curative resection? (3) Does the use of adjuvant therapy affect outcome?

## Patients and methods

A retrospective review was conducted of a prospectively maintained database to identify patients with a histological diagnosis of dedifferentiated CS managed at a single tertiary musculoskeletal oncology centre. Minimum follow-up was 12 months or until death. 116 patients were diagnosed with dedifferentiated CS between 1995 and 2016.

**Fig. 1:**
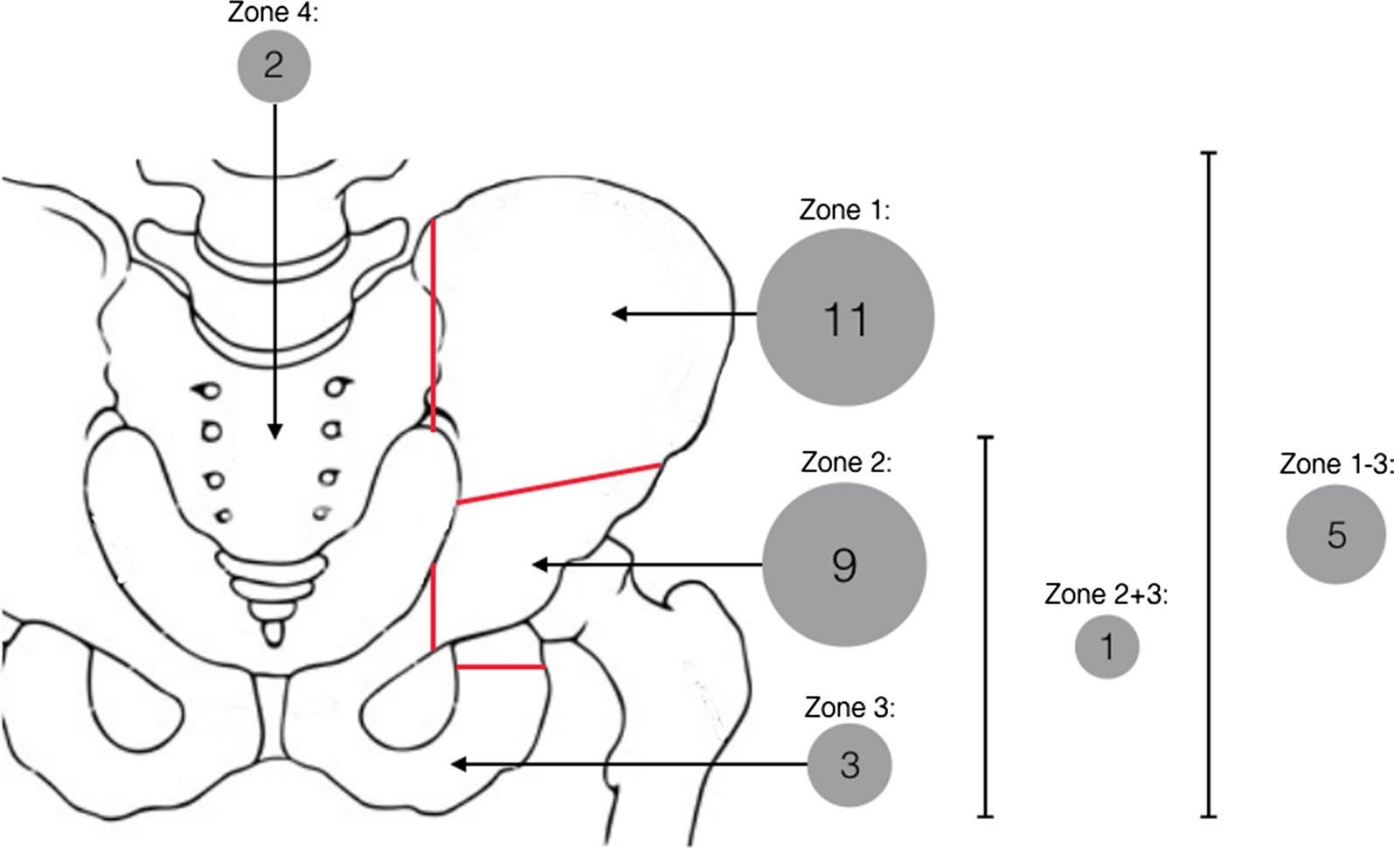
Frequency of tumour location within the pelvis

31 patients were identified with pelvic dedifferentiated CS. They had a mean age of 55.6 years (range 33 to 76 years). There were 19 males (61.3%) and 12 females (38.7%). The ilium (P1) was involved in 51.6% (16/31) of tumours. 48.4% of tumours (15/31) involved the periacetabular (P2) region either in isolation or in combination with other pelvic regions (Fig. 1).

Local tumour staging comprised of plain radiography and magnetic resonance imaging (MRI). Distant staging comprised of chest computed tomography (CT) and bone scintigraphy. All patients were managed by a specialist sarcoma multidisciplinary team (MDT). Histological diagnosis was based on biopsy material and reviewed by a specialist sarcoma pathologist prior to surgical treatment. In 11/18 patients (61.1%) undergoing surgical resection the histological diagnosis of a dedifferentiated chondrosarcoma was made on the biopsy material pre-operatively. In the remaining seven patients (38.9%) pre-operative biopsy failed to accurately grade the tumour when compared to the post resection histology. Of these seven patients, three had a biopsy that suggested a low-grade CS, two a high-grade CS and in two, a spindle cell sarcoma with no cartilaginous elements (Table 1).

**Table 1:** Patients demographics and outcomes who underwent attempted curative resection

Patient	Age	Sex	Surgery	Margin	Correct pre-op diagnosis	Time to LR	Time to metastases	Time to death	Time alive	Status
1	61	F	AMPHQ	Intralesional	Yes	14	13	33		DOD
2	62	M	AMPHQ	Wide	Yes				121	NED
3	52	F	AMPHQ	Marginal	Yes	4	3	4		DOD
4	55	M	AMPHQ	Wide	Yes				51	NED
5	55	F	AMPHQ	Wide	Yes				12	NED
6	72	M	LSS	Intralesional	No			0		DOD
7	51	M	LSS	Marginal	No	2	2	5		DOD
8	49	M	LSS	Intralesional	Yes	10	12	12		DOD
9	43	F	LSS	Marginal	Yes	13	13	15		DOD
10	71	M	LSS	Marginal	Yes		2	4		DOD
11	47	M	LSS	Intralesional	No	5	5	9		DOD
12	71	F	LSS	Marginal	No		2	3		DOD
13	44	F	LSS	Wide	No	20	24	29		DOD
14	63	F	LSS	Wide	Yes				100	NED
15	47	F	LSS	Marginal	No				63	NED
16	57	F	LSS	Intralesional	Yes	3	0	7		DOD
17	48	M	LSS	Intralesional	No	2	11		24	AWD
18	54	M	LSS	Intralesional	Yes	2	2	3		DOD

*F* female, *M* male, *AMPHQ* hindquarter amputation, *LSS* limb salvage surgery, *DOD* dead of disease, *NED* no evidence of disease, *AWD* alive with disease

**Fig. 2:**
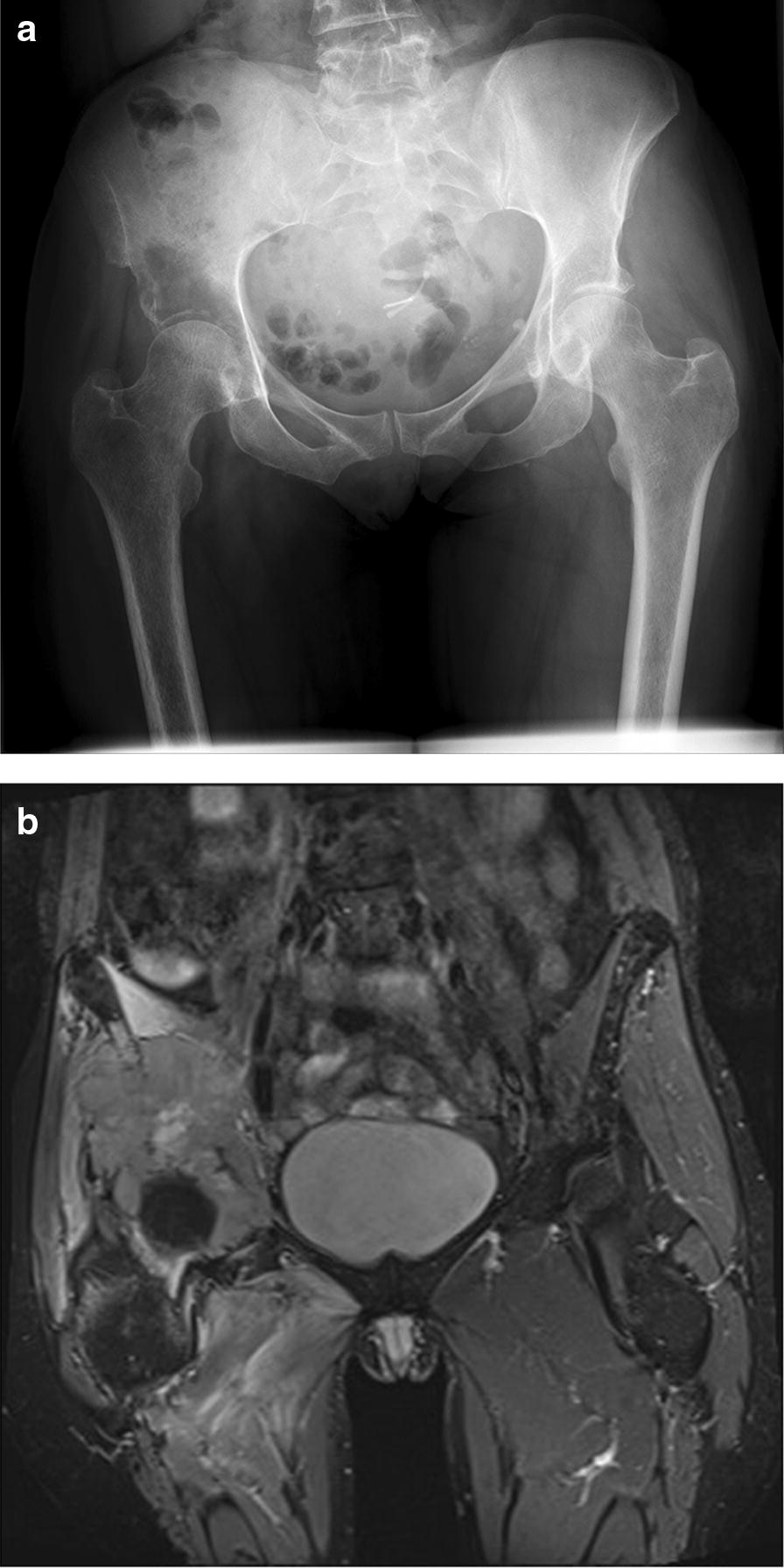
Pre-operative pelvis radiograph (**a**) and MRI (**b**) of a patient who underwent HQA. In this case, there was a large tumour involving both the hip joint and femoral neurovascular bundle

**Fig. 3:**
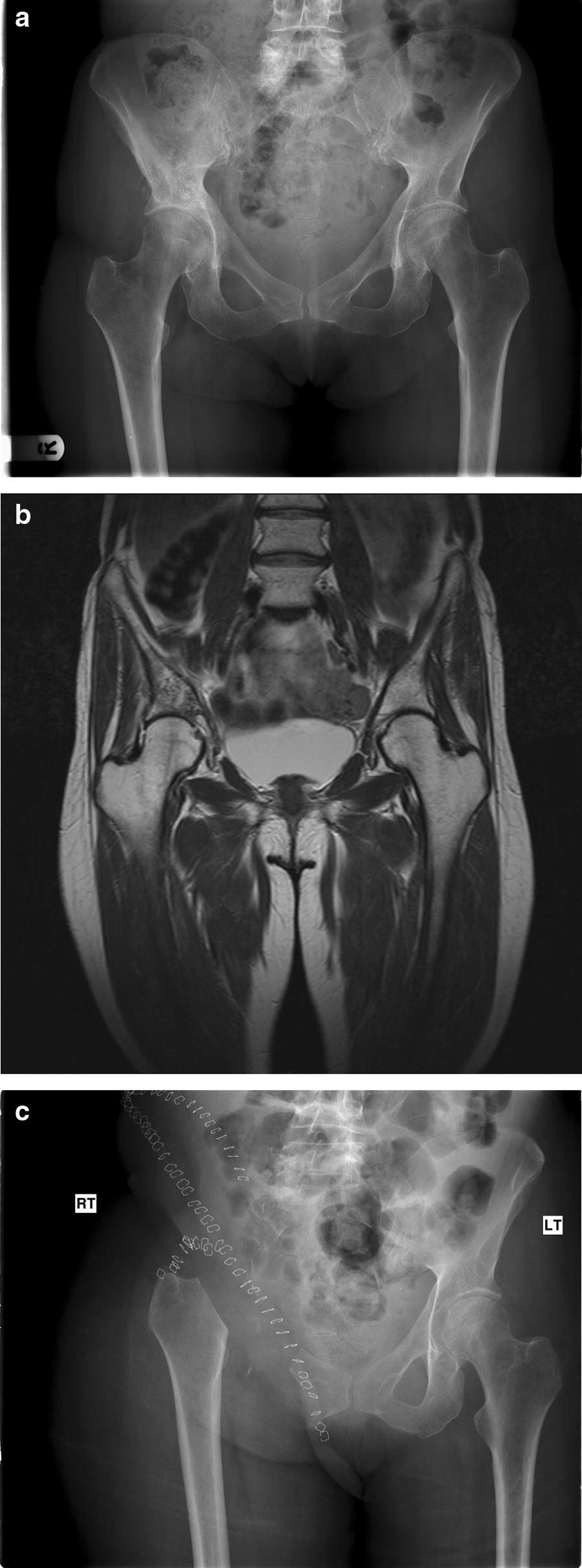
Pre-operative pelvis radiograph (**a**) and MRI (**b**) and post-operative radiograph (**c**) of a patient who underwent LSS through an internal hemipelvectomy and no reconstruction (flail hip) with wide margins (> 20 mm) and long-term survival achieved

Wide margins, the aim of surgery, were defined as greater than 4 mm on final pathological specimen analysis [11]. Indications for hindquarter amputation (HQA) were involvement of two of the following three structures: sciatic nerve, hip joint, external iliac vessels or where it was deemed the safest way to achieve wide margins (Figs. 2a, b, 3a–c). Chemotherapy and radiotherapy was guided by oncologists on an individualised basis to attempt systemic or local short-term tumour control.

Patients were divided into palliative or surgical treatment groups. 13 patients (41.9%) were in the palliative group. Patients were not offered surgery due to either advanced metastatic disease (in 10 patients), the tumour was deemed unresectable (entire hemipelvis involvement, including the sacrum and sciatic notch), too medically unfit for surgery or died during neoadjuvant chemotherapy (one patient each) (Table 2). Patients in the surgical group were analysed according to the surgical procedure and surgical margins.

**Table 2:** Patient demographics by treatment group

	Overall cohort	Limb-salvage (LSS)	Hindquarter amputation (HQA)	Palliative	p-value (between LSS and HQA groups)
Patient number (n, %)	31	13 (41.9%)	5 (16.1%)	13 (41.9%)	
Age (mean, range)	55.6 (33–76)	55.2 (43–72)	57 (52–62)	55.5 (33–76)	0.716
Enneking stage (n, %)					
Stage 2b	21 (67.7%)	13 (100%)	5 (100%)	3 (23.1%)	1.000
Stage 3b (metastases)	10 (32.3%)	0	0	10 (76.9%)	1.000
Biopsy diagnosis correct (n, %)	11 (61.1%)	6 (46.1%)	5 (100%)	N/A	0.036
Tumour location, solitary P1 lesions (n, %)	11 (35.5%)	1 (7.7%)	3 (60%)	7 (53.8%)	0.017
Tumour size (max diameter, cm)	110.9 (45–200)	108.8 (45–200)	117.5 (90–180)	N/A	0.756
Chemotherapy (n, %)	15 (48.4%)	6 (46.1%)	2 (40%)	7 (53.8%)	0.814
Radiotherapy (n, %)	7 (22.6%)	2 (15.4%)	1 (20%)	4 (30.8%)	0.814
Surgery complications	2 (11.8%)	2 (15.4%)	0	N/A	0.352

18 patients (58.1%) underwent attempted curative treatment by surgery. 13 patients underwent limb-salvage surgery (LSS) and five HQA. None of the patients in the surgical group had metastases at the time of diagnosis. For those treated by LSS, six (46.2%) had internal hemipelvectomies without reconstruction, three (23.1%) had a stemmed acetabular prosthesis (“ice-cream” cone), three (23.1%) had custom implants and one (7.7%) had combined internal hemipelvectomy and a proximal femoral replacement.

One patient died from a post-operative cardiac arrest and another suffered a periprosthetic infection which was successfully treated through debridement, implant retention and antibiotics.

Statistical analysis was performed using R and deducer statistical software packages, and considered statistically significant at p < 0.05. Analysis of local recurrence-free survival (LRFS), metastasis-free survival (MFS), disease-free (DFS) and overall survival (OS) was performed using the Kaplan–Meier survival method with 95% confidence intervals (CI). To assess the effect of different factors on survival outcomes, the Cox proportional hazards (PH) model was used.

## Results

### Palliative group

41.9% of patients (n = 13) were considered incurable by resection of the primary tumour and were offered palliative treatment only. In this group, the median survival was 3 months (IQR 2 to 8 months); two patients (15.4%) in this group survived beyond 12 months. The longest survival (34 months), was seen in a patient treated with palliative radiotherapy alone as they were unfit for ablative surgery.

### Curative group

**Fig. 4:**
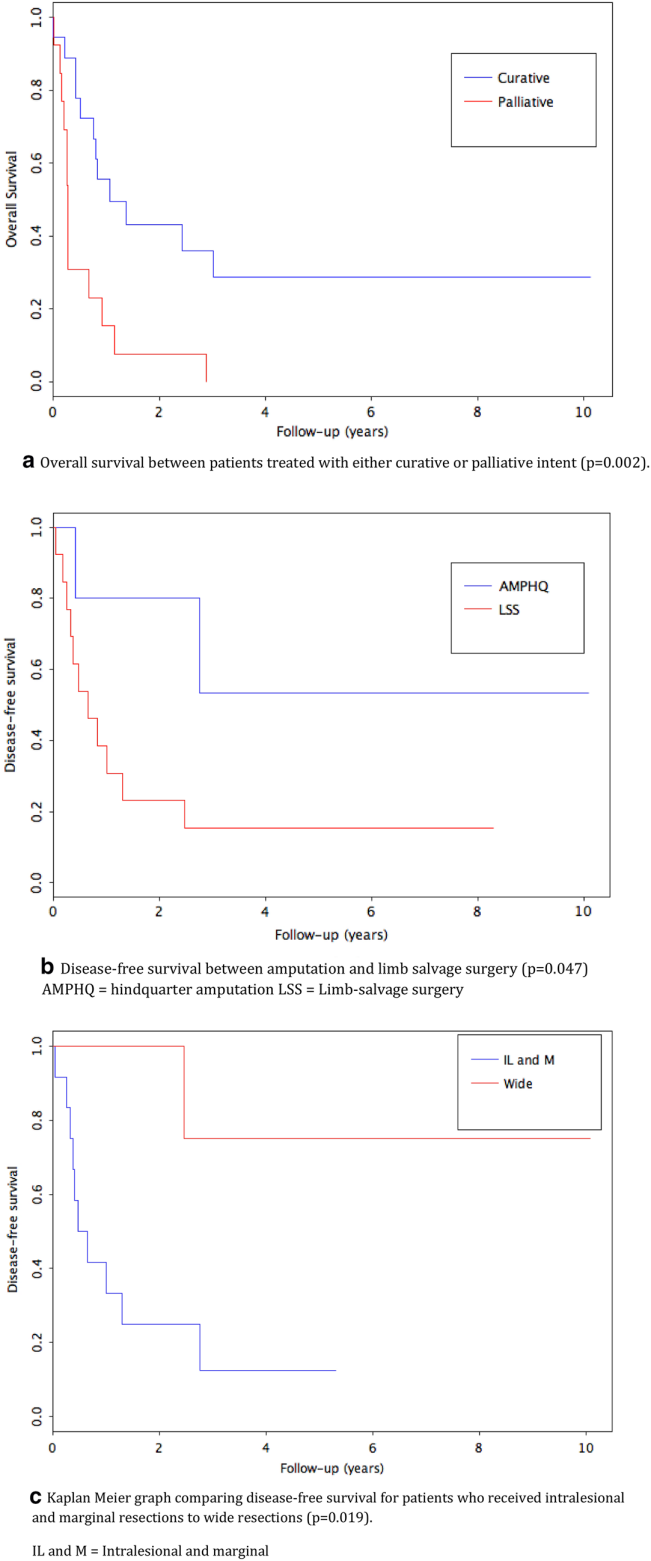
**a** Overall survival between patients treated with either curative or palliative intent (p = 0.002). **b**. Disease-free survival between amputation and limb-salvage surgery (p = 0.047). AMPHQ = hindquarter amputation; LSS = Limb-salvage surgery. **c**. Kaplan–Meier graph comparing overall survival for patients who received intralesional and marginal resections to wide resections (p = 0.019). *IL* intralesional, *M* marginal

Of the patients (n = 18, 58.1%) who underwent attempted curative resection with wide margins, the OS at 12 months was 50.0% (95% CI 31.5%–79.4%), at 36 months was 29.2% (95% CI 13.4%–63.4%) (Fig. 4a). At 12 months, the LRFS and MFS were 56.1% (95% CI 36.1%–87.2%) and 47.2% (95% CI 28.6%–78.1%), respectively. At 36 months, the LRFS and MFS were 32.7% (95% CI 15.2%–70.2%) and 28.3% (95% CI 13.0%–61.7%), respectively. The mean time to local recurrence and metastasis was 7.5 months (range 2 to 20 months) and 7.4 months (range 0.5 to 24 months), respectively. There were five patients with long-term DFS (27.8%) after undergoing surgery with curative intent. There was no difference in age (p = 0.842), size (p = 0.191) or proportion of isolated P1 area tumours (p = 0.260) between survivors and those with disease recurrence or death.

### Limb-salvage versus amputation

Of the five patients who underwent HQA, three (60%) are still alive with no evidence of disease at 12, 51 and 121 months follow-up. All three had wide margins of excision. The remaining two patients (40%) had marginal and intralesional excision margins and both developed local recurrence (4 and 14 months, respectively), metastases (3 and 13 months, respectively) and died (4 and 33 months, respectively) (Table 1).

Of the 13 patients who underwent LSS, three patients remain alive at a mean 62.3 months since operation, of which, two have no evidence of disease, at 100 and 63 months, one had a wide and the other a marginal excision, respectively. The remaining 10 patients have died (one dying in perioperative period from cardiac arrest). The LRFS and MFS at last follow-up was 22.2% (95% CI 6.7%–73.8%) and 16.8% (95% CI 4.7%–59.4%), respectively. The mean time to local recurrence was 7.1 months (range 2 to 20 months), mean time to metastases was 7.3 months (range 0 to 24 months) and the mean time to death was 8.7 months (range 0 to 29 months).

The DFS for the LSS cohort was 15.4% (95% CI 4.3%–55.0%) and 60% for the HQA group. The DFS was significantly greater in patients who underwent HQA (p = 0.047) (Fig. 4b). There was no significant difference between groups in time to local recurrence or metastases (p = 0.776 and p = 0.914, respectively).

90% of patients with recurrence developed local recurrence and metastases synchronously (maximum 4 months between events). One patient developed local recurrence at 2 months with metastases detected at 11 months.

3/13 (23%) patients in the LSS group underwent secondary HQA for locally recurrent disease. The mean time from surgery to additional operation was 11.7 months. A wide resection margin at subsequent HQA was achieved in two patients and a marginal margin in one. One patient died from a complication of the amputation, one lived an additional 9 months and the other remains alive with local and systemic disease 21 months following HQA.

### Margins

The type of surgery significantly affected the margins achieved. HQA achieved wide margins more frequently than LSS (p = 0.05) (Table 3).

**Table 3:** Surgical margins achieved by the operation conducted

Surgical margins	Overall	Limb salvage	Hindquarter amputation	Secondary hindquarter amputation
Intralesional (n,%)	7 (33.3%)	6 (46.1%)	1 (20.0%)	0
Marginal (n, %)	7 (33.3%)	5 (38.5%)	1 (20.0%)	1 (33.3%)
Wide (n, %)	7 (33.3%)	2 (15.4%)	3 (60.0%)	2 (66.7%)

Disease-free survival was greater in patients who received wide resection margins (p = 0.019). Of the five patients presumed cured, with no evidence of disease at last follow-up, four (80%) had wide resection margins, the last had marginal margins. Overall, 80% of patients who had wide margins were cured, 16.7% of patients with marginal margins were cured and no patients with intralesional margins were disease-free at last follow-up (Table 1 and Fig. 4c).

### Chemotherapy and radiotherapy

Chemotherapy was not used routinely, but was used palliatively or postoperatively following the development of recurrent disease. In most cases where chemotherapy was used, it consisted of cisplatin and doxorubicin. One patient had neoadjuvant chemotherapy, 4 cycles of cisplatin and doxorubicin, after the initial biopsy suggested the diagnosis of spindle cell sarcoma. Following resection, dedifferentiated CS was confirmed. This patient had < 10% necrosis, a marginal margin and is alive at 63 months following surgery (being the only survivor with a marginal margin).

Radiotherapy was used palliatively or for management of local recurrence. The radiotherapy dose ranged from 30 to 70 Grays depending on adjacent anatomical structures, tumour site and size. No patients who had radiotherapy survived.

## Discussion

In all chondrosarcomas, survival correlates with histological grade, and the dedifferentiated CS subtype is known to confer the worst prognosis [1, 9, 10, 12]. In accordance with other studies, dedifferentiated CS has a peak incidence in the 6th decade of life, a male predilection, and a male-to-female ratio of 3:2 observed in our series [12–14].

Dedifferentiated CS has been associated with a poor prognosis [10]. The best chance of cure was linked to excision of the tumour with clear margins [10]. Achieving clear margins is much more difficult in the pelvis, even with HQA. The role of chemotherapy and radiotherapy is largely palliative [7, 8, 15–18]. The presenting clinical scenario and treatment of pelvic dedifferentiated CS has not been specifically examined previously, however, data extrapolated from previous studies clearly reveals pelvic disease confers a dreadful prognosis (Table 4) [5, 9, 10, 12, 19, 20].

**Table 4:** Survival outcomes for all patients with dedifferentiated CS of the pelvis treated with either palliative or curative intent from available literature over the last 30 years

Study	Number of patients	Mortality rate	Time to disease recurrence, months	Notes
Frassica et al. [5]	23	19–21 (82.6%–91.3%)	N/A	Calculated from available data (pelvic data not explicitly reported)
Liu et al. [9]	13	12 (92.3%)	10.6	
Grimer et al. [10]	95	N/A	N/A	No pelvis survival outcomes reported
Mavrogenis et al. [12]	32	22 (68.8%)	13	Calculated from available data on Kaplan–Meier curves
Weber et al. [19]	1	1 (100%)	8	
Sheth et al. [20]	13	10 (76.9%)	4.5	
Current study	31	25 (80.6%)	6.2	

In the present study concerning dedifferentiated CS of the pelvis, 42% of patients presented with such advanced disease that surgery was not a viable treatment option. Unsurprisingly, patients able to undergo surgery with curative intent had greater overall survival than the palliative group. The only long-term survivors with no evidence of disease (16.1% of the cohort) underwent attempted curative surgical resection with wide or marginal margins.

Patients who underwent primary HQA had higher rates of disease-free survival than those who underwent LSS, 60% vs 15.4%, respectively. This likely correlated to the higher rates of wide margins achieved with HQA. In patients who had LSS, 66.7% had local recurrence and in the those who did not develop local recurrence, two are long term survivors and the other two died at 3 and 4 months. Other studies of predominantly non-dedifferentiated pelvic CS have revealed satisfactory margins can be achieved with limb-salvage [6, 12, 13]. However, one study identified a significantly higher chance of obtaining a clear margin with amputation [21]. Our data suggests that wide margins are more achievable with HQA, which was translated into improved local control and survival. The primary operation to consider in those with dedifferentiated CS should be a HQA with wide margins to offer patients the best chance of cure. In this series, none of the three patients who had HQA with wide margins died, compared to only three survivors in the 15 patients who had any other combination of surgery and margin type.

Obtaining a wide margin has been reported to be a positive prognostic indicator for non-dedifferentiated CS [11, 14, 22]. However, it remains unclear whether achieving a wide margin around dedifferentiated CS impacts local recurrence [12–14, 23]. In our series, there was an 80% chance of achieving disease cure when wide margins, greater than 4 mm, were obtained. Of the five patients who were completely disease-free at last follow-up, four had a wide margin and the other a marginal margin. There were no long-term survivors in those with intralesional tumour resection.

Another issue with pelvic dedifferentiated CS, reflecting the tumour heterogeneity and large volume, is that 7 of the 18 curative patients did not have the correct diagnosis on initial biopsy. Three of these patients were thought to have low-grade CS and LSS was carried out with very close margins. Although one died postoperatively the other two both developed recurrent disease at 2 months. This highlights the importance of obtaining wide margins in any pelvic CS. Four of the five patients who were disease-free at last follow-up had accurate pre-operative biopsies. Saifuddin et al. have suggested taking biopsies from areas of reduced signal intensity on T2-weighted MRI may result in higher detection rates of dedifferentiated CS [24].

For isolated local recurrence of dedifferentiated CS treated with amputation, no patients achieved a cure or long-term survival (mean survival = 9.7 months). LR and metastases occurred synchronously in 90%. A study reporting surgical outcomes for recurrent CS of the pelvis also showed no overall survival advantage when treating recurrent high-grade CS tumours [19]. Therefore, the priority should be initial curative resection to reduce the chance of recurrence as rapid disease progression, with subsequent death can be anticipated.

In our series, one of the notable findings was that all recurrent disease, whether local or systemic arose within 24 months. This is likely a reflection of the extremely aggressive nature of this tumour. A high chance of cure can be expected if a patient lacks any evidence of disease after 24 months follow-up. A short time to recurrent disease was also evident in previous studies (Table 4).

We found no benefit for chemotherapy or radiotherapy used in a palliative setting, reaffirming the belief that chemotherapy and radiotherapy are ineffective at controlling dedifferentiated CS [7].

The limitations to this series are that the data is based on a retrospective analysis from a single institution, with inherent selection bias both in those treated surgically and in the type of surgical procedure. This study was not able to analyse whether having wide margins from the dedifferentiated component of the tumour or if having a small proportion of dedifferentiation within the tumour was associated with increased survival as this was not routinely reported by pathologists, however, these would be important areas to research in the future. Additionally, the sample size was small, reflecting the rarity of this disease, therefore whether amputation is truly superior at achieving radical section margins cannot be proven, and a larger multi-institutional review would help clarify this.

## Conclusion

Dedifferentiated CS of the pelvis confers a poor prognosis. 12-month survival is 15.4% in those treated palliatively and 55.6% when treated with curative intent. It is a particularly aggressive disease, presenting at an advanced, inoperable state in almost half of patients. The factors that influenced disease cure were achieving a wide surgical margin (greater than 4 mm), which was more common with HQA. Margins less than 4 mm have a very high risk of local recurrence and death. HQA for local recurrence did not result in disease control and 90% of disease recurrence occurred synchronously. Obtaining the correct pre-operative diagnosis is also an important factor. We recommend early surgery with the widest possible margins to optimise chances for long-term survival.

## Abbreviations

CS: chondrosarcoma
MRI: magnetic-resonance imaging
CT: computed tomography
MDT: multidisciplinary team
HQA: hindquarter amputation
LSS: limb-salvage surgery
LRFS: local recurrence-free survival
MFS: metastasis-free survival
DFS: disease-free survival
OS: overall survival
CI: confidence interval
